# Re-evaluating Cardiovascular Risk: A Narrative Review Challenging the Cholesterol Hypothesis and Identifying Modern Dietary Drivers

**DOI:** 10.7759/cureus.105311

**Published:** 2026-03-16

**Authors:** Mitchell B Liester, Jenna D Moore

**Affiliations:** 1 Psychiatry, University of Colorado School of Medicine, Colorado Springs, USA; 2 Nutrition, Summit Performance Nutrition LLC, Colorado Springs, USA

**Keywords:** apolipoprotein b (apob), cardiovascular risk assessment, diet-heart hypothesis, lipoprotein(a), refined carbohydrates and metabolic syndrome, saturated fat and cholesterol, trans fatty acids, triglyceride-to-hdl ratio

## Abstract

Between 1920 and 1950, cardiovascular disease (CVD) underwent a profound epidemiological shift, rising from a relatively rare and infrequently diagnosed condition to become the leading cause of death in industrialized nations. This epidemic coincided with a series of changes in the food supply, including the expanded use of refined carbohydrates, industrial seed and vegetable oils, and trans fatty acids. In response, the "Diet-Heart Hypothesis" emerged, dominated by Ancel Keys' lipid theory, which focused scientific and public health attention on saturated fat and cholesterol as the primary causes of CVD. This paradigm profoundly shaped dietary guidelines for decades, yet the sugar industry's documented influence on nutritional research during this period raises questions about how economic interests may have deflected scrutiny from other dietary factors. This review critically examines the evolution of cardiovascular risk assessment, exploring both the historical context of CVD emergence and the contemporary evidence supporting biomarkers that may be better at predicting risk than traditional cholesterol-focused approaches. Significant evidence reveals limitations in the lipid hypothesis, which oversimplified cardiovascular risk by demonizing total and LDL cholesterol. Research now demonstrates that apolipoprotein B and non‑HDL cholesterol more accurately reflect atherogenic lipoprotein burden than LDL cholesterol alone, while the triglyceride‑to‑HDL cholesterol ratio is a useful marker of insulin resistance and metabolic dysfunction. Lipoprotein(a), an independent genetic risk factor, accounts for a substantial proportion of cardiovascular events previously attributed to other causes. Furthermore, inflammatory markers like high-sensitivity C-reactive protein add prognostic value beyond traditional lipid panels. Perhaps most importantly, the historical dominance of saturated fat as a dietary "villain" is challenged by contemporary meta-analyses showing no significant association with CVD, while the roles of refined carbohydrates, industrial trans fats, and excess omega-6 fatty acids, such as those in soybean oil, warrant greater scrutiny. Contemporary cardiovascular risk assessment must move beyond LDL cholesterol-centric approaches to incorporate comprehensive metabolic and inflammatory markers. Apolipoprotein B, lipoprotein(a), triglyceride-to-HDL ratio, and high-sensitivity C-reactive protein provide more nuanced risk stratification, while dietary recommendations should acknowledge that industrial food processing, refined carbohydrates, and specific fatty acid compositions may pose greater cardiovascular threats than naturally occurring saturated fats. This paradigm shift demands updated clinical guidelines that reflect current scientific understanding rather than historical assumptions, potentially revolutionizing both prevention and treatment strategies for CVD.

## Introduction and background

The dramatic rise in coronary heart disease (CHD) deaths during the early 20th century represents one of the most consequential epidemiological transitions in modern medicine. In the early decades of that century, CHD was relatively uncommon and infrequently diagnosed. By mid-century, however, it had become the leading cause of death in the United States, surpassing traditional infectious diseases such as tuberculosis and pneumonia, whose mortality rates were declining due to improvements in sanitation and, later, the advent of antibiotics [1]. By the 1950s, myocardial infarction had reached epidemic proportions, particularly among middle-aged men. This substantial rise in CHD mortality over only a few decades cannot be explained by population aging alone and likely reflects a combination of changes in smoking, diet, blood pressure, physical activity, and improved recognition and reporting of myocardial infarction [2,3].

Identifying the dietary and metabolic drivers of this transition has proven considerably more difficult than documenting its epidemiological contours. Multiple hypotheses have been proposed and contested over the past seven decades, and the evidence base continues to evolve. What is well-established is that CHD risk is not uniformly distributed across populations and that broad shifts in diet, lifestyle, and industrialized food production have accompanied the rise in disease burden, although the precise causal contributions of individual factors remain a matter of active scientific debate.

This narrative review examines the evolution of cardiovascular disease (CVD) risk assessment by exploring both the historical context of dietary and lipid hypotheses and the contemporary evidence supporting biomarkers that may better predict CVD risk than traditional cholesterol-focused approaches. The review aims to distinguish between areas of established scientific consensus, emerging evidence, and hypotheses that remain plausible but not yet definitively proven.

Methods

A literature search was conducted in PubMed/MEDLINE, Embase, and Google Scholar using terms related to CVD epidemiology, the diet-heart and lipid hypotheses, emerging atherogenic biomarkers (apolipoprotein B, lipoprotein(a), LDL particle number, triglyceride-to-HDL ratio, high-sensitivity C-reactive protein), and dietary cardiovascular risk factors (saturated fat, trans fatty acids, refined carbohydrates, seed oils). Articles selected for inclusion were those written in English and published between 1950 and 2025, with priority given to randomized controlled trials, systematic reviews, and meta-analyses.

## Review

The modern era brought about a combination of environmental and lifestyle shifts that set the stage for this health crisis. Traditional diets, which were rich in home-cooked meat, fish, eggs, and natural fats, were systematically displaced by the outputs of the burgeoning industrialized food complex. This new diet consisted predominantly of ultra-processed foods loaded with refined sugars, enriched flours, and industrial seed oils [1-4]. The consumption of these new, chemically refined ingredients occurred simultaneously with a widespread decrease in physical activity due to the automation of labor and reliance on automobiles [1,5,6]. These parallel trends created a perfect physiological storm, challenging metabolic systems that had not evolved to cope with such highly refined and industrially processed ingredients.

While the initial epidemic primarily affected middle-aged and often affluent men in the 1950s, later epidemiological data, particularly after 1970, revealed that the greatest risk of CHD and early death disproportionately fell upon the poorest and long-term unemployed segments of society [2]. We hypothesize that this distribution pattern suggests that the basic drivers of chronic disease are linked to the cheapest, most ubiquitous products of the industrialized food system - refined carbohydrates and high-polyunsaturated fatty acid (PUFA) seed oils - thereby accelerating disease in the most vulnerable populations. Table 1 provides a timeline of key historic events.

**Table 1:** A Century of Cardiovascular Disease: The Rise, the Paradigm, and the Re-evaluation. Key Historical Events, 1913–2024 Timeline of key events from 1913 to 2024. CHD = coronary heart disease; SRF = Sugar Research Foundation; SCS = Seven Countries Study; LDL-C = low-density lipoprotein cholesterol; hs-CRP = high-sensitivity C-reactive protein; TFA = trans fatty acid; RCT = randomized controlled trial.

Year	Category	Event/Development	Description/Impact
1913	Major Studies & Evidence	Anitschkow: Theoretical Basis of the Lipid Hypothesis	Nikolai Anitschkow establishes the theoretical basis for the lipid hypothesis, linking dietary cholesterol to atherosclerosis in animal models.
1922	Major Studies & Evidence	de Langen: Fat-Rich Diet Raises Cholesterol 27%	Cornelis de Langen demonstrates that Indonesians placed on a fat-rich "Dutch diet" experience an average serum cholesterol rise of 27%, providing early human evidence for the diet-cholesterol link.
1920s–1930s	CVD Rise & Mortality	CHD Surpasses Infectious Disease as Leading U.S. Cause of Death	Coronary heart disease rapidly escalates to become the #1 cause of death in the U.S., surpassing tuberculosis and pneumonia.
1950	CVD Rise & Mortality	CHD Reaches Epidemic Proportions	Heart attacks reach epidemic proportions, particularly among middle-aged men. The sudden rise between 1922 and 1950 cannot be attributed to longer lifespans alone, implicating novel dietary or environmental factors.
1953	Major Studies & Evidence	Ancel Keys Formally Proposes the Lipid-Heart Hypothesis	Keys theorizes that high total cholesterol—from dietary saturated fat and cholesterol—drives fat deposition into arterial walls, causing atherosclerosis and heart disease.
1954	Processed Food & Industry	Sugar Research Foundation Identifies Financial Motive	The Sugar Research Foundation (SRF) documents that low-fat diets would cause sucrose consumption to "increase by more than one-third," establishing a clear profit motive to promote the low-fat paradigm.
1955	Dietary Guidelines & Policy	Eisenhower's Heart Attack Cements the Fat Hypothesis in Policy	On September 23, President Eisenhower suffered a heart attack; his physician, Dr. Paul Dudley White, publicly urged Americans to cut fat and cholesterol, creating an "unassailable chain of male authority" for the lipid hypothesis.
1956	Major Studies & Evidence	First Evidence That Trans Fats Increase CAD Risk	Early evidence suggests industrial trans fatty acids can increase coronary artery disease risk—a finding largely overlooked for three decades.
1958	Major Studies & Evidence	Seven Countries Study Formally Launched	Keys begins his landmark multinational study comparing diet and lifestyle across cohorts in seven countries, linking saturated fat intake to CHD mortality.
Mid-1960s	Processed Food & Industry	Sugar Industry Funds Harvard Literature Reviews	The SRF directs Harvard researchers to produce reviews that "single out fat and cholesterol" as dietary causes of CHD and downplay evidence linking sucrose to cardiovascular risk.
1977	Dietary Guidelines & Policy	U.S. Senate Issues Dietary Goals for Americans	The U.S. Senate Select Committee formally recommends low-fat, low-cholesterol diets for all Americans—the starting point of the modern U.S. dietary guideline era.
1991-2007	Dietary Guidelines & Policy	Denmark's Trans Fat Ban Reduces CHD Deaths ~11%	Denmark's ban on industrial trans fatty acids is associated with an approximately 11% reduction in CHD deaths, providing direct evidence of TFAs' pathological role.
Post-1990s	Reform & Re-Evaluation	Research Undermines Dietary Cholesterol Restriction	Accumulating clinical and epidemiological evidence demonstrates that dietary cholesterol has only a minimal impact on serum cholesterol levels for most people, undermining the scientific basis of the original dietary restrictions.
2008	Major Studies & Evidence	JUPITER Trial: Inflammation as Independent CVD Target	The JUPITER trial demonstrates that statin therapy reduces cardiovascular events in individuals with elevated hs-CRP (>2.0 mg/L) but normal LDL-C, providing evidence that targeting inflammation independently reduces CVD risk.
2010	Reform & Re-Evaluation	Siri-Tarino et al.: No Association Between Saturated Fat & CVD	A meta-analysis of 21 prospective cohort studies (347,747 subjects) finds no significant association between dietary saturated fat and risk of CHD, stroke, or CVD—directly challenging four decades of dietary guidance.
2015	Dietary Guidelines & Policy	2015 Dietary Guidelines Remove Quantitative Cholesterol Limit	For the first time since 1961, the 2015-2020 Dietary Guidelines for Americans officially remove the specific 300 mg/day dietary cholesterol cap, as policy catches up to scientific consensus.
2016	Reform & Re-Evaluation	Kearns et al.: Sugar Industry Manipulation Documented	Publication of internal SRF documents in JAMA Internal Medicine confirms the sugar industry's deliberate manipulation of nutritional science—paying researchers to deflect scrutiny from sucrose onto saturated fat.
2024	Reform & Re-Evaluation	Yamada et al.: Saturated Fat Reduction Not Recommended	A systematic review and meta-analysis of 9 RCTs (13,532 participants) finds no significant differences in CVD mortality, all-cause mortality, or MI between saturated fat reduction and control groups.

The role of trans fatty acids

The introduction of partially hydrogenated oils containing industrial trans fatty acids represents another critical dietary change during this period. Trans fats were created through the industrial process of partial hydrogenation of vegetable oils, which increased shelf life and improved the texture of processed foods [7]. Evidence as early as 1956 suggested that trans fats could increase coronary artery disease, with studies in the early 1990s bringing renewed scrutiny and confirmation of their negative health impact [8,9].

Trans fatty acids increase CVD risk through multiple mechanisms. They raise LDL cholesterol while simultaneously lowering HDL cholesterol. They also increase inflammatory markers, impair endothelial function, and promote insulin resistance [10,11]. A 2% increase in energy intake from trans fats has been associated with a 23% increase in cardiovascular risk [12,13]. Epidemiological studies demonstrate that individuals in the highest quartile of trans fat consumption have nearly triple the risk of CHD compared to those in the lowest quartile, even after adjusting for other risk factors [14]. Denmark's ban on trans fats led to an ~11% reduction in CHD deaths from 1991 to 2007, providing evidence of their pathological role [15].

This distribution pattern suggests that the foundational drivers of chronic disease risk are linked to the cheapest, most ubiquitous products of the industrialized food system - refined carbohydrates and high-polyunsaturated fatty acid (PUFA) seed oils - thereby accelerating disease in the most vulnerable populations.

Ancel Keys' lipid-heart hypothesis

In the search for a culprit to explain the explosion of heart disease, scientists quickly focused on cholesterol and dietary fat. The theoretical basis for what later became known as the "Lipid Hypothesis" was established as early as 1913 by Nikolai Anitschkow (also spelled Anichkov), whose cholesterol‑feeding experiments in rabbits produced atherosclerotic lesions resembling those seen in humans [16]. Early controlled work by Cornelis de Langen in the Dutch East Indies showed that Javanese laborers consuming a traditional, low‑fat, rice‑based diet had much lower serum cholesterol than Europeans, and that when Javanese subjects were shifted to a fat‑rich “Dutch diet” high in animal fats, their serum cholesterol rose substantially, by roughly 40 mg/dL on average in his small trial [17]. Over the 20th century, the intake of omega‑6‑rich industrial vegetable oils, including soybean oil, rose sharply in the United States, while the availability of traditional animal fats such as butter and lard declined [18]. Building upon this concept, physiologist Ancel Keys formally proposed his lipid-heart hypothesis in 1953. Keys theorized that high total cholesterol, resulting from consuming excessive saturated fat and cholesterol, increased fat deposition into arterial walls, thereby driving atherosclerosis and heart disease [19].

In the mid‑1950s, Keys began international surveys that culminated in the formally launched Seven Countries Study (SCS) in 1958. This study compared diet and lifestyle factors across cohorts in seven countries (i.e., Japan, Greece, the U.S., Italy, the Netherlands, Serbia, and Finland), linking high saturated fat intake with CHD mortality [19].

The SCS immediately drew intense scrutiny and criticism concerning its methodology. Detractors alleged that Keys cherry-picked data, claiming he studied 22 countries but published only the seven that supported his hypothesis. Proponents of Keys argue that there were "never more than seven countries involved in the study" [20]. Similarly, the exclusion of France, known for the "French Paradox" - high saturated fat consumption coupled with low rates of heart disease - was frequently cited as a sign of manipulation. However, Keys' defenders clarified that French researchers were invited but declined participation [20,21].

Importantly, the debate extended to confounding dietary factors. Critics asserted that Keys overlooked or even buried data suggesting sugar had a stronger association with CHD than saturated fat. While Keys' defenders contend that he specifically addressed sugar intake in the SCS but found a stronger association with saturated fat, the overall environment was one where fat was pre-emptively identified as the primary risk factor [20,21]. This pattern suggests a research environment in which the initial correlation was elevated to a causal law, thereby inhibiting deeper inquiry into highly correlated but less popular risk factors, such as refined carbohydrates and sugars.

This hypothesis gained rapid institutional prominence in the mid‑20th century. When U.S. President Dwight Eisenhower suffered a heart attack in September 1955, his cardiologist, Paul Dudley White, helped elevate Ancel Keys’ diet‑heart hypothesis into the national spotlight by publicly emphasizing diet - especially saturated fat and cholesterol - as key drivers of CHD. Their combined authority contributed to the broad acceptance of low‑fat dietary advice among clinicians and policymakers, even as some researchers, such as British nutritionist John Yudkin, continued to argue that sugar and refined carbohydrates were more important causes of heart disease [22].

The Sugar Council and the blame shift

The widespread adoption of the fat-as-villain hypothesis created a significant financial opportunity for competing food interests. Internal documents from the sugar industry reveal a calculated strategy aimed at capitalizing on the low-fat trend. The trade organization, the Sugar Research Foundation (SRF), recognized as early as 1954 that if Americans adopted low-fat diets, the consumption of sucrose would likely "increase by more than one-third" [23]. This document provides clear evidence of a powerful profit motive driving the promotion of a low-fat dietary paradigm.

By the mid-1960s, the sugar industry actively began working with nutrition scientists at Harvard to shape the dominant narrative. Documents show that the SRF collaborated with researchers, paying them to produce literature reviews that would "single out fat and cholesterol as the dietary causes of coronary heart disease" and, conversely, "downplay evidence that sucrose consumption was also a risk factor.” In one instance, the level of cooperation was such that the industry instructed the Harvard researchers on which competing papers they were "unhappy with" and to "deal with them" in their forthcoming reviews [23]. This manipulation demonstrates the subversion of the peer-review process, wherein industry funding was used to manufacture scientific consensus in influential academic publications, effectively derailing objective inquiry into sugar’s true role in cardiovascular pathology.

It is important to acknowledge the historical nuance regarding the nature of this influence. While the documents clearly show industry funding and direction [23], some historians argue that the sugar industry merely exploited a pre-existing academic bias that already favored the fat hypothesis, rather than initiating a total "sugar conspiracy" [24]. Regardless of the moral intent behind the collaboration, the practical result was the institutionalization of dietary guidelines based on skewed or incomplete data, leading directly to the proliferation of high-sugar, low-fat processed foods.

While the sugar industry was shaping public perception, the scientific understanding of cholesterol metabolism was itself evolving in ways that would ultimately challenge the lipid hypothesis.

The scientific evolution of cholesterol metabolism

Decades of subsequent clinical and epidemiological research have gradually dismantled the central premise of the early diet-heart hypothesis: the idea that consuming dietary cholesterol directly and significantly elevates serum cholesterol for the general population.

The human body possesses sophisticated mechanisms of cholesterol homeostasis, primarily regulated by the liver. When dietary intake of cholesterol increases, internal production tends to decrease, maintaining a stable plasma equilibrium for most individuals. The preponderance of clinical and epidemiological data accumulated since the original dietary restrictions were formulated consistently indicates that dietary cholesterol has only a minor impact on plasma cholesterol levels in the majority of the population [25]. However, there is substantial inter-individual variability in response to dietary cholesterol, driven by differences in both intestinal cholesterol absorption efficiency and hepatic synthesis regulation. A subset of individuals - termed "hyper-responders" - exhibit clinically meaningful increases in plasma LDL cholesterol following dietary cholesterol loading, while "hypo-responders" demonstrate little to no change [26]. Accordingly, population-level conclusions regarding the modest effect of dietary cholesterol should not be applied uniformly to all individuals, and clinical guidance may need to account for a patient's individual metabolic phenotype.

A meta-analysis by Berger and colleagues (2015) reported that added dietary cholesterol significantly increases both LDL cholesterol (the traditionally pathologized particle) and HDL cholesterol (the cardioprotective particle), and it also found a small, statistically significant increase in the LDL:HDL ratio (net change around 0.2). This ratio is a far more significant predictor of heart disease risk than LDL cholesterol alone. Numerous epidemiological surveys have confirmed the lack of a significant relationship between cholesterol intake and heart disease incidence [25].

Despite the overwhelming accumulation of this evidence, changing the official dietary recommendation proved to be a slow and at times consuming process. This institutional inertia, which resulted in part from cognitive dissonance, meant that public health guidelines remained based on an invalidated hypothesis for a half-century, potentially creating negative consequences, such as inadequate intake of essential nutrients like choline, often found in high-cholesterol foods like eggs [25].

The genesis of modern American dietary guidelines began in 1977 with the U.S. Senate Select Committee’s report Dietary Goals for the United States, which recommended low‑fat, low‑cholesterol diets for everyone [27]. This is widely viewed as the starting point of the modern U.S. guideline era [28]. The recommendations to limit saturated fat and cholesterol remained remarkably consistent for nearly 40 years [28], even as the scientific foundation crumbled. The official removal of the quantitative limit on dietary cholesterol finally occurred in 2015 with the publication of the 2015-2020 Dietary Guidelines for Americans [29].

Having examined the historical factors that shaped the lipid hypothesis, we now turn to the contemporary evidence that challenges its foundational assumptions.

Contemporary re-evaluation of the saturated fat hypothesis

Recent meta-analyses and systematic reviews have significantly challenged the foundational premise of the diet-heart hypothesis. A 2010 meta-analysis of 21 prospective epidemiologic studies involving 347,747 subjects found no association between dietary saturated fat intake and increased risk of CHD, stroke, or CVD [30]. The pooled relative risk estimates comparing extreme quantiles of saturated fat intake were 1.07 (95% CI: 0.96-1.19) for CHD and 0.81 (95% CI: 0.62-1.05) for stroke, neither of which reached statistical significance.

A 2020 systematic review examining randomized controlled trials concluded that meta-analyses of observational studies found no association between saturated fat intake and heart disease, while meta-analyses of randomized controlled trials were inconsistent but tended to show a lack of association [31]. Most compellingly, a very recent 2024 systematic review and meta-analysis of nine randomized controlled trials with 13,532 participants found no significant differences in cardiovascular mortality (RR=0.94, 95% CI: 0.75-1.19), all-cause mortality (RR=1.01, 95% CI: 0.89-1.14), myocardial infarction (RR=0.85, 95% CI: 0.71-1.02), or coronary artery events (RR=0.85, 95% CI: 0.65-1.11) between saturated fat reduction and control groups [32]. These findings led the authors to conclude that "a reduction in saturated fats cannot be recommended at present to prevent cardiovascular diseases and mortality."

The accumulating evidence suggests that the health effects of foods cannot be predicted by their saturated fat content alone without considering the overall macronutrient distribution and food matrix. Whole-fat dairy, unprocessed meat, and other saturated fat-rich foods with complex matrices are not associated with increased cardiovascular disease risk when consumed as part of balanced dietary patterns [33].

Rancid seed oils and endothelial toxicity

**Figure 1:**
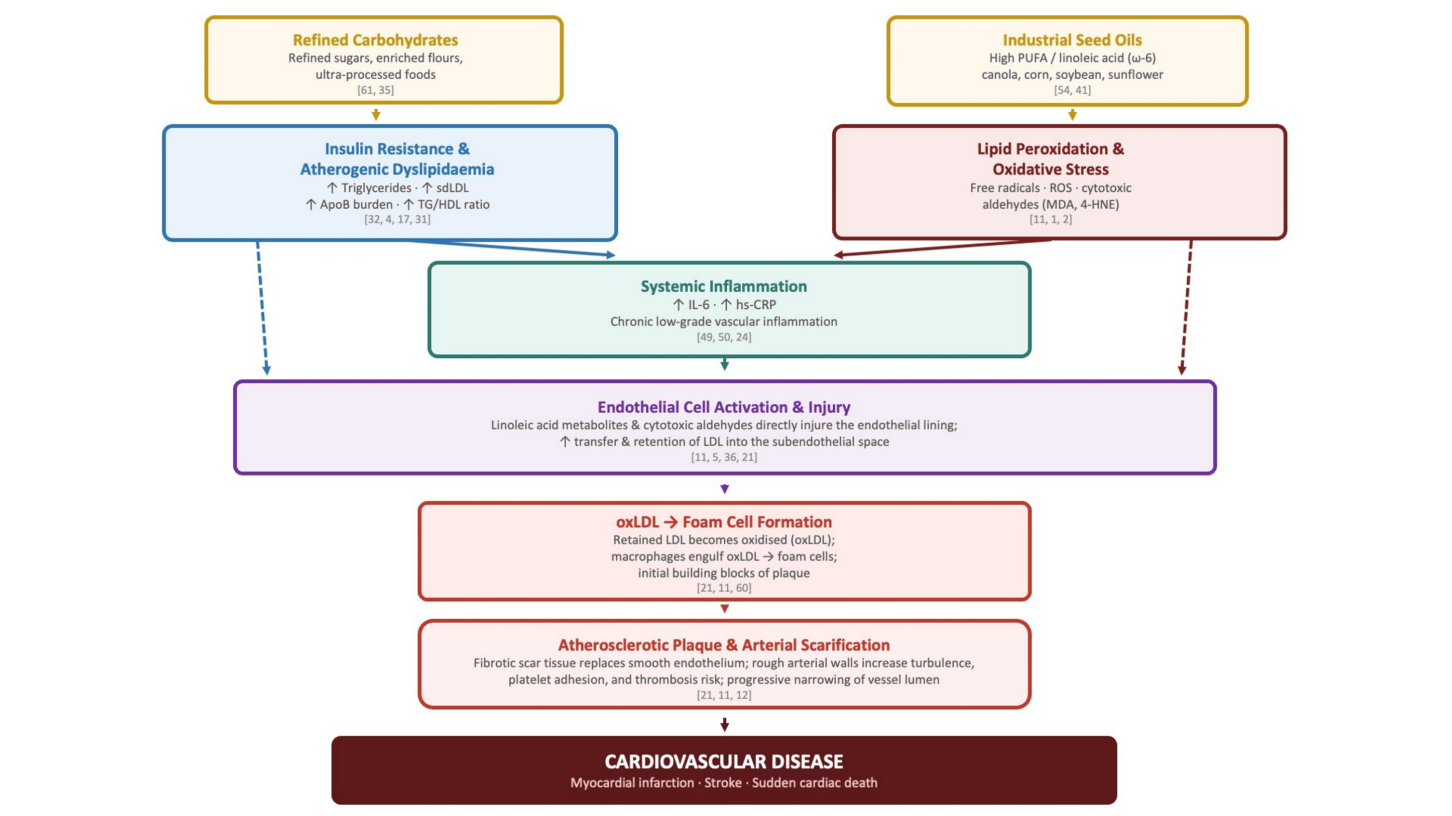
Mechanistic Pathways From Dietary Factors to Atherosclerosis Mechanistic pathways from dietary factors to atherosclerotic cardiovascular disease. Two primary dietary inputs are shown. The carbohydrate/metabolic pathway (blue): refined carbohydrates promote insulin resistance and atherogenic dyslipidemia, characterized by elevated triglycerides, small dense LDL (sdLDL), increased ApoB particle burden, and an elevated TG/HDL-C ratio [4,17,31,32]. The seed oil/oxidative pathway (red): industrial seed oils high in polyunsaturated fatty acids (PUFAs), particularly linoleic acid (ω-6), undergo lipid peroxidation, generating reactive oxygen species (ROS) and cytotoxic aldehydes—malondialdehyde (MDA) and 4-hydroxy-2-nonenal (4-HNE) [1,2,11]. Both pathways contribute to systemic inflammation (↑ IL-6, ↑ hs-CRP) [24,50,49] and converge on endothelial cell injury, increasing LDL transfer into the subendothelial space. Retained LDL is oxidized (oxLDL), triggering macrophage engulfment and foam cell formation—the initial building blocks of atherosclerotic plaque [11,21]. Progressive plaque accumulation and arterial scarification culminate in cardiovascular disease. Dashed arrows indicate direct pathway contributions that bypass the inflammatory node. 4-HNE = 4-hydroxy-2-nonenal; ApoB = apolipoprotein B; hs-CRP = high-sensitivity C-reactive protein; MDA = malondialdehyde; oxLDL = oxidized LDL; PUFA = polyunsaturated fatty acid; ROS = reactive oxygen species; sdLDL = small dense LDL; TG/HDL-C = triglyceride-to-HDL cholesterol ratio. Image created by the authors using MS PowerPoint (Microsoft Corporation, Redmond, Washington).

While early dietary guidelines emphasized reducing saturated fat and cholesterol, more recent research also highlights the importance of overall dietary patterns, including the quality and processing of fats and carbohydrates (Figure 1). Diets high in ultra‑processed foods, rich in refined carbohydrates, and omega‑6-rich vegetable oils have been associated with systemic inflammation, oxidative stress, and metabolic dysfunction, particularly when omega‑6 intake greatly exceeds omega‑3 intake. Drawing on biochemical reasoning and clinical observation, Dr. Catherine “Cate” Shanahan has argued in popular books and commentaries that highly processed industrial seed oils (“vegetable oils”) are a major driver of modern chronic disease through mechanisms involving oxidative stress and inflammation, although this view is not a settled scientific consensus [34].

The mechanism of lipid peroxidation and free radical cascades

The key to the pathology of seed oils lies in their high content of polyunsaturated fatty acids (PUFAs), especially linoleic acid (LA), a type of omega-6 fatty acid. These molecules are highly vulnerable to degradation because their multiple double bonds are chemically unstable [18]. Industrial processing, which uses chemical solvents and refining methods, and the subsequent application of heat during cooking - particularly repeated heating, as often occurs in commercial frying - initiate a process known as lipid peroxidation [35].

This peroxidation process generates highly toxic free radicals and reactive oxygen species (ROS) that induce severe oxidative stress at the cellular and molecular levels. The degradation of these oxidized lipids produces cytotoxic aldehydes, notably malondialdehyde (MDA) and 4-hydroxy-2-nonenal (4-HNE) [36]. It is important to note that much of this evidence derives from in vitro and animal studies, as well as biochemical modeling; direct causal evidence from controlled human clinical trials remains limited and is an active area of ongoing investigation.

These reactive compounds act as "second messengers" of oxidative stress, interacting with various macromolecules, including DNA, proteins, and phospholipids, causing widespread molecular damage [37]. While this mechanistic pathway is biochemically well-characterized and biologically plausible, the extent to which these processes translate into clinically meaningful cardiovascular outcomes in human populations has not yet been definitively established. The available human data, including observational studies and biomarker analyses, are consistent with the proposed mechanism but fall short of confirming direct causation. Distinguishing between mechanistic plausibility and demonstrated causal effect in living populations remains a critical and unresolved challenge in this field, and readers should interpret the foregoing biochemical evidence accordingly.

Endothelial damage and arterial scarification

The vascular endothelium, which is the single-cell layer lining the inside of all blood vessels, is the primary target of these chemically aggressive lipid peroxidation products. Linoleic acid and its metabolites have been shown to activate and inflame vascular endothelial cells, initiating a critical step in the development of atherosclerosis [18].

The body registers the damage caused by these oxidized compounds as a physical injury, prompting a pathological repair mechanism. Lipid peroxidation products directly injure the endothelial cells, thereby increasing the transfer and retention of LDL into the vessel wall [18]. Once retained in the subendothelial space, the LDL itself becomes oxidized (oxLDL), attracting immune cells (macrophages), which consume the oxLDL and become foam cells, which are the initial building blocks of atherosclerotic plaque.

Under this model, atherosclerosis is viewed fundamentally as a misguided healing response, in which inflammation and subsequent fibrotic repair (scarification) attempt to patch the oxidized damage [38]. Instead of maintaining the healthy, smooth, non-thrombogenic surface required for optimal blood flow, this scar tissue replaces the sophisticated endothelial lining. This process results in the arterial walls becoming rough and abrasive, conceptually akin to internal pipes lined with sandpaper rather than being smooth, thereby increasing turbulence, platelet adhesion, and the risk of clot formation [18].

It is important to note that this hypothesis conflicts with conventional nutrition recommendations, which often promote seed oils because they are effective at reducing LDL cholesterol levels compared to saturated fats [39]. However, the argument, supported by Shanahan and the supporting literature, is that the quality of the particle (its oxidative state) and the damage it inflicts on the arterial wall are far more critical than the sheer cholesterol mass it carries. Numerous lines of evidence show that the increased consumption of omega-6 PUFAs promotes oxidative stress, oxidized LDL, chronic inflammation, and atherosclerosis, potentially increasing the risks of CHD and death despite any observed reduction in total LDL-C [18].

The role of systemic inflammation

Beyond lipid parameters, chronic low-grade systemic inflammation plays a central role in the pathogenesis of atherosclerotic cardiovascular disease. High-sensitivity C-reactive protein (hs-CRP) has emerged as the most widely validated biomarker for assessing cardiovascular inflammation and predicting future cardiovascular events. CRP is produced by the liver in response to pro-inflammatory cytokines, particularly interleukin-6 (IL-6), and elevated levels reflect ongoing vascular inflammation even in apparently healthy individuals [40].

Numerous epidemiological studies have demonstrated that elevated hs-CRP levels predict incident myocardial infarction, stroke, peripheral arterial disease, and sudden cardiac death among individuals with no history of cardiovascular disease [40,41]. Meta-analyses of observational studies show that individuals in the top quartile for hs-CRP levels have an odds ratio of approximately 1.5 for major cardiovascular events compared to those in the lowest quartile, even after adjusting for traditional risk factors [41]. The landmark JUPITER trial demonstrated that statin therapy reduced cardiovascular events in individuals with elevated hs-CRP (>2.0 mg/L) but normal LDL cholesterol levels, providing evidence that targeting inflammation independently reduces cardiovascular risk [42].

Clinical guidelines now recognize hs-CRP as a "risk-enhancing factor" for CVD risk assessment. Values less than 1.0 mg/L indicate low cardiovascular risk, 1.0-3.0 mg/L indicate moderate risk, and greater than 3.0 mg/L indicate high risk [43]. The integration of hs-CRP measurement with advanced lipid biomarkers provides a more comprehensive assessment of residual inflammatory risk, particularly in patients whose LDL cholesterol is optimally controlled but who remain at elevated risk for cardiovascular events.

Accurate markers for cardiovascular risk

The reliance on LDL-C as the primary determinant of cardiovascular risk is increasingly recognized as a clinical deficiency. While standard guidelines continue to recommend strict LDL-C targets, LDL-C measures the mass of cholesterol contained within the LDL particles, failing to account for the actual number of circulating particles or their relative toxicity. Evidence indicates that despite a large percentage of heart disease patients not meeting LDL-C targets, clinicians often fail to modify or intensify treatment accordingly [44]. More accurate and mechanistically relevant biomarkers exist for assessing true atherogenic risk (Table 2).

**Table 2:** Summary of Advanced Cardiovascular Biomarkers ApoB = apolipoprotein B; hs-CRP = high-sensitivity C-reactive protein; TG/HDL-C = triglyceride-to-HDL cholesterol ratio; LDL-C = low-density lipoprotein cholesterol; VLDL = very low-density lipoprotein; IDL = intermediate-density lipoprotein; ASCVD = atherosclerotic cardiovascular disease

Biomarker	Clinical Utility	Reference Ranges	Limitations	Additional Clinical Notes
ApoB (Apolipoprotein B)	Direct count of total atherogenic lipoprotein particles (VLDL, IDL, LDL) capable of penetrating the endothelial lining and initiating plaque formation. Superior to LDL-C for predicting ASCVD, especially when LDL-C and non-HDL-C are discordant. Recommended as a primary clinical measure for estimating risk and evaluating lipid-lowering therapy.	Optimal: <80 mg/dL (low-risk) Borderline: 80–100 mg/dL Elevated: >100 mg/dL (Lower targets recommended for high-risk patients)	Not universally included in standard lipid panels; requires a specific assay. Less familiar to many clinicians than LDL-C. Cost may limit widespread adoption. Does not distinguish particle size.	Elevated ApoB with normal LDL-C (discordance) confers high cardiovascular risk. Represents the total burden of dangerous particles, making it the most mechanistically relevant single lipid biomarker.
Lp(a) (Lipoprotein(a))	Independent, causal genetic risk factor for ASCVD and aortic valve stenosis. Adds risk beyond LDL-C, even with statin therapy. Guidelines recommend measuring at least once in adults, especially those with premature CVD, family history, or familial hypercholesterolemia.	Desirable: <30 mg/dL Borderline: 30–50 mg/dL High risk: >50 mg/dL (~80th percentile) 2–3× increased MI risk above threshold	70–90% genetically determined; unaffected by diet, exercise, or most lipid-lowering medications. No widely approved Lp(a)-lowering therapies currently available (agents in clinical trials). Assay standardization varies across labs.	Multifactorial atherogenesis: promotes endothelial dysfunction, LDL oxidation, pro-inflammatory effects, and prothrombotic activity (structural homology with plasminogen). Accounts for residual CVD risk in statin-treated patients.
LDL-P (LDL Particle Number)	Total count of circulating LDL particles; superior to LDL-C for risk assessment. Detects 'hidden' cardiovascular risk in patients with normal LDL-C but elevated particle count (e.g., on statin therapy). Strongly correlated with systemic insulin resistance, inflammation, hypertriglyceridemia, and coronary artery calcium score.	Optimal: <1000 nmol/L Near-optimal: 1000–1299 nmol/L Borderline: 1300–1599 nmol/L High: ≥1600 nmol/L (by NMR spectroscopy)	Requires specialized NMR spectroscopy or ion mobility testing; less widely available than standard lipid panels. Higher cost. Closely correlated with ApoB—simultaneous testing may be redundant. Less familiar in routine clinical practice.	Small, dense LDL (sdLDL) subtype is more atherogenic: penetrates subendothelial space more easily and is more susceptible to oxidation and retention. High sdLDL is a hallmark of insulin resistance.
TG/HDL-C (Triglyceride-to-HDL Ratio)	Simple, cost-effective surrogate marker for insulin resistance and metabolic syndrome. Reflects hepatic overproduction of TG-rich lipoproteins, reduced lipoprotein lipase activity, and decreased reverse cholesterol transport. Independently predicts incident CVD, type 2 diabetes, and metabolic syndrome.	Optimal: <2.0 Borderline: 2.0–3.0 Insulin resistant/high risk: >3.0–3.5 (mg/dL units; multiply by ~0.45 for mmol/L)	Ethnic variability: African Americans may have normal triglycerides despite insulin resistance, limiting utility. Triglycerides are highly variable (fasting state, diet, alcohol). Does not directly measure particle number or quality.	Highly predictive in Caucasian, Hispanic, and Asian populations. Elevated ratio associated with small, dense LDL, higher BP, elevated fasting glucose, increased inflammatory markers, and greater carotid intima-media thickness.
hs-CRP (High-Sensitivity C-Reactive Protein)	Most widely validated biomarker for cardiovascular inflammation. Liver-derived in response to IL-6; elevated levels reflect vascular inflammation even in apparently healthy individuals. Recognized as a 'risk-enhancing factor' in clinical guidelines. The JUPITER trial showed statin therapy reduced CVD events in patients with elevated hs-CRP but normal LDL-C.	Low risk: <1.0 mg/L Moderate risk: 1.0–3.0 mg/L High risk: >3.0 mg/L (Values >10 mg/L suggest acute infection/injury; repeat testing recommended)	Non-specific: elevated by any systemic inflammation (infection, autoimmune disease, obesity, smoking). Does not identify the source of inflammation. A single elevated value may reflect acute illness rather than chronic cardiovascular risk.	Individuals in the top quartile for hs-CRP have ~1.5× odds ratio for major CVD events vs. the lowest quartile. Provides additive prognostic value beyond lipid panels, especially for residual inflammatory risk in LDL-C-optimized patients.

ApoB

ApoB is a protein segment found on the surface of every single atherogenic lipoprotein, including VLDL (very low-density lipoprotein), IDL (intermediate-density lipoprotein), and LDL. Measuring ApoB provides a direct count of the total number of circulating particles capable of penetrating the endothelial lining and initiating plaque formation [44]. Atherosclerosis is fundamentally a disease of particle burden; the greater the number of circulating ApoB-containing particles, the higher the likelihood of a particle entering the arterial wall.

Studies using discordance analysis, where ApoB levels conflict with LDL-C or non-HDL-C levels, provide strong evidence that ApoB is a significantly more accurate marker of cardiovascular risk than either LDL-C or non-HDL-C [44,45]. For instance, when ApoB is elevated but LDL-C is within the target range, the risk of cardiovascular events remains high [46]. Consequently, ApoB should serve as the primary clinical measure to estimate risk and evaluate the effectiveness of lipid-lowering therapies [44].

Lp(a)

Lp(a) represents one of the most significant genetic cardiovascular risk factors, affecting approximately 20-25% of the global population [47]. Lp(a) is an LDL-like particle with an additional apolipoprotein(a) component covalently bound to apolipoprotein B-100. Unlike other lipid parameters, Lp(a) levels are 70-90% genetically determined and remain relatively stable throughout life, unaffected by diet, exercise, or most lipid-lowering medications [48].

Extensive epidemiological and Mendelian randomization studies have established elevated Lp(a) as an independent, causal risk factor for atherosclerotic CVD and aortic valve stenosis [49,50]. Individuals with Lp(a) levels above 50 mg/dL (approximately the 80th percentile) face a 2-3-fold increased risk of myocardial infarction and cardiovascular events, comparable to the risk associated with familial hypercholesterolemia [50]. Importantly, elevated Lp(a) confers increased cardiovascular risk even when LDL cholesterol is well-controlled with statin therapy, contributing to residual risk.

The atherogenic mechanisms of Lp(a) are multifactorial, including promotion of endothelial dysfunction, facilitation of LDL oxidation through associated oxidized phospholipids, pro-inflammatory effects, and prothrombotic activity due to structural homology with plasminogen [51]. Current guidelines recommend measuring Lp(a) at least once in adults to identify those at increased risk, particularly individuals with premature cardiovascular disease, a family history of elevated Lp(a), or familial hypercholesterolemia [52]. While specific Lp(a)-lowering therapies are currently in clinical trials, identifying elevated Lp(a) allows for aggressive management of other modifiable risk factors.

LDL-P and size

Closely related to ApoB, the measurement of LDL-P is also considered a superior tool for risk assessment and a better indicator of subclinical disease than conventional LDL-C. Elevated LDL-P correlates with systemic inflammation, insulin resistance, hypertriglyceridemia, and an increased coronary artery calcium score, reflecting the underlying metabolic damage caused by high intake of sugars and processed foods [53-55].

Furthermore, LDL-P measurement detects hidden cardiovascular risk, especially in patients whose LDL-C values appear low, including those on statin therapy [56]. When the particle count is high, even if the cholesterol load (LDL-C) is low, the risk remains substantial.

Particle size also plays a significant role. Small, dense LDL (sdLDL) particles are structurally more dangerous because they penetrate the vascular subendothelial space more easily and are more susceptible to oxidation and retention [57]. High levels of sdLDL are a hallmark of insulin resistance, indicating that the pathology originates from the body's inability to process refined carbohydrates and oxidized fats effectively [58,59].

Triglyceride/HDL cholesterol ratio (TG/HDL-C)

The TG/HDL-C has emerged as a simple, cost-effective surrogate marker for insulin resistance and metabolic syndrome, both of which are powerful drivers of CVD [60]. This ratio reflects the underlying metabolic dysfunction that accompanies insulin resistance, including increased hepatic production of triglyceride-rich lipoproteins, reduced lipoprotein lipase activity, and decreased reverse cholesterol transport.

Multiple studies have demonstrated strong correlations between elevated TG/HDL-C ratios and insulin resistance as measured by the gold-standard hyperinsulinemic-euglycemic clamp or HOMA-IR [59]. In Caucasian populations, a TG/HDL-C ratio greater than 3.0-3.5 effectively identifies insulin-resistant individuals at increased cardiometabolic risk [60]. Elevated TG/HDL-C ratios are associated with increased prevalence of sdLDL particles, which are more atherogenic than large, buoyant LDL [61].

Prospective studies have shown that the TG/HDL-C ratio independently predicts incident cardiovascular events, type 2 diabetes, and metabolic syndrome [62,63]. A cross-sectional analysis demonstrated that individuals with elevated TG/HDL-C ratios had significantly worse cardiometabolic risk profiles, including higher blood pressure, fasting glucose, inflammatory markers, and carotid intima-media thickness [64]. The ratio's predictive value for cardiovascular disease appears comparable to or superior to traditional metrics like LDL-C alone, particularly for identifying individuals with insulin resistance-driven atherosclerosis.

It is important to note that the TG/HDL-C ratio shows ethnic variability in its relationship with insulin resistance. While highly predictive in Caucasian, Hispanic, and Asian populations, African Americans may have normal triglycerides despite insulin resistance, potentially limiting the ratio's utility in this population [65]. Nevertheless, for routine clinical practice, the TG/HDL-C ratio provides readily available information about metabolic health and cardiovascular risk that extends beyond traditional lipid measures.

The nuance of the cholesterol paradox

LDL-C alone is an incomplete measure of cardiovascular risk. While certain studies correlate high dietary cholesterol intake with increased MI risk [66], these findings must be interpreted in light of the overall lipid profile. The widely reported phenomenon that some populations with elevated LDL-C and total cholesterol levels may have a decreased risk of heart disease is often explained by lipoprotein particle composition: when LDL is predominantly composed of large, buoyant particles accompanied by high levels of protective HDL, cardiovascular risk is lower. The true danger lies not in cholesterol volume but in the number and quality (oxidation, small size) of ApoB-containing particles present, confirming that ApoB and LDL-P are superior metrics for identifying pathological risk.

Synthesis and comprehensive timeline

The historical understanding of cholesterol’s role in CVD is a story of initial epidemiological correlation being mistakenly elevated to causation, reinforced by political authority and exploited by commercial interests. This suggests that the cardiovascular epidemic that began in the 1920s may have been driven by the introduction of novel dietary elements, such as refined sugars and industrial seed oils, which create systemic metabolic dysfunction (insulin resistance) and direct cellular damage (oxidative stress/free radical cascades). The result is a high burden of chemically modified, highly atherogenic lipid particles. The focus must shift from simply lowering LDL-C mass to reducing the particle count and improving particle quality, measured by advanced biomarkers.

## Conclusions

The historical role of cholesterol in CVD, which was propagated by institutional inertia and corporate influence, did not fully capture the complexity of the root causes of the modern cardiovascular epidemic. The dominant pathology is best captured by the burden of ApoB‑containing lipoprotein particles rather than LDL‑C mass alone. Chronic endothelial injury caused by oxidative stress and metabolic damage resulting from excessive refined sugar consumption leads to an overproduction of highly atherogenic ApoB-containing particles. Thus, excessive intake of refined sugar is a significant contributor to CVD. The role of industrial seed oils in the pathogenesis of CVD also warrants further investigation.

Clinical practice could be improved by adding ApoB (or non-HDL-C) and LDL-P to accurately quantify the true atherogenic particle burden and to improve risk assessment. Dietary recommendations should be adjusted to minimize refined sugar intake to reduce oxidative stress and inflammation, thereby decreasing the risk of CVD. Additional research is needed to assess the effects of limiting industrial seed oils, particularly in highly processed foods, to determine whether this will further reduce CVD risk.
